# Online Star vs. Celebrity Endorsements: The Role of Self-Concept and Advertising Appeal in Influencing Purchase Intention

**DOI:** 10.3389/fpsyg.2021.736883

**Published:** 2021-11-23

**Authors:** Pengfei Shi, Xiaojing Lu, Yi Zhou, Chaojing Sun, Liying Wang, Biao Geng

**Affiliations:** ^1^School of Economics and Management, Southwest University, Chongqing, China; ^2^School of Economics and Management, Wuhan University, Wuhan, China; ^3^Business School, Hubei University, Wuhan, China; ^4^Department of Business Administration, Shandong Labor Vocational and Technical College, Jinan, China; ^5^China Academy of Civil Aviation Science and Technology, Beijing, China

**Keywords:** celebrity endorsement, online star endorsement, self-concept, purchase intention, advertising appeal

## Abstract

Despite the fact that companies increasingly value online star endorsements as Internet celebrity economy booms, scientific knowledge on the effect of online star endorsements on consumers’ purchase intention is limited. Based on the theories of self and construal level theory, this study investigates the impact of online star vs. celebrity endorsements on purchase intention and explores the underlying mechanism as well as boundary conditions. The results of four studies reveal the following: (1) Compared with no endorsement, both celebrity endorsements and online star endorsements lead to increases in consumers’ purchase intention, with no significant difference between the two. (2) Self-concept mediates these relationships; specifically, celebrity and online star endorsements activate the ideal and actual self respectively, and enhance consumers’ willingness to purchase. (3) The effect of endorsements on consumers’ purchase intention is moderated by advertising appeals. That is, celebrity endorsements enhance purchase intention when consumers are exposed to symbolic appeals in advertisements, and online star endorsements enhance purchase intention when it is matched with functional advertising appeals.

## Introduction

Online stars, also known as Internet celebrities (*wang hong* in Chinese) are becoming most popular with Internet users due to the rapid expansion of social media platforms, such as YouTube, Facebook, Sina Weibo, Tik Tok, and Kuaishou. While social celebrities remain popular influencers in mainstream markets and are highly valued by companies, online stars have increasingly begun to be deployed as endorsers, for example, Bella Hadid endorsing Nike, and Papi Jiang and Zhang Dayi advertising Weibo Story. Unlike traditional celebrities, online stars often thrive in ordinary social groups like common consumers, who perceive a sense of affinity and closeness with these stars (Stever and Lawson, 2013; Clarke et al., 2016; Zhang et al., 2020).

Although many academic studies have confirmed the economic value of various endorser types (Ayeh, 2015), little is known about the effectiveness of celebrity vs. online star endorsements. While researches have focused on the development of online stars, measurement of their influence, and relationships between online stars and fans (Abidin and Thompson, 2012; Clarke et al., 2016), few have examined the effect of online stars and traditional celebrities on purchase intention. Further, as businesses increasingly rely on social media platforms to promote their products, consumers are changing their purchasing habits, their state of mind, cognition or perception (Lăzăroiu et al., 2020). Thus, their self-concept and purchase intention are also influenced (Verwijmeren et al., 2011; Krishna, 2012; Vrontis et al., 2021). Although the underlying processes of endorser effect have been explained by the source-attractiveness model, meaning transfer theory, matching theory, or elaboration likelihood model (Erdogan, 1999; Erdogan and Baker, 2000; Kerr et al., 2015; Zamudio, 2016), little is known about the role of the activated self-concept—either the ideal self or actual self—on consumers’ purchase intention. What’s more, substantial literature has both categorized advertising appeals, such as rational and emotional appeals, hard-selling and soft-selling appeals, thought and sensory appeals, and symbolic and functional appeals (Shavitt, 1992; Gauzente and Roy, 2012; Lantos, 2015), and discussed the moderating effects of these appeals on various factors like consumers, cultures, products, channels, and celebrities (Chang, 2006; Choi et al., 2012; Vazifehdoust et al., 2014; Kunkel et al., 2018). However, research has remained largely dedicated to matching symbolic and functional appeals with different product categories but has failed to match them with celebrity and online star endorsements.

To address this research gap, the current study focuses on the effects of celebrity endorsements and online star endorsements on consumers’ purchase intention. Specifically, we address the following questions: (1) What is the effect of online star endorsement (vs. no endorsement) on purchase intention? Is there a significant difference between the effects of celebrity endorsement and online star endorsement on purchase intention? (2) What is the psychological mechanism underlying the effects of these two types of endorsements? (3) How are these effects moderated by symbolic and functional advertising appeals?

## Theory and Hypotheses

### Online Stars and Social Celebrities

Online stars, also called cyberspace stars, web celebrities, online celebrities, or web influencers, are usually focal figures who attract numerous fans by creating and enthusiastically sharing original texts, pictures, or videos on social media (Stever and Lawson, 2013). They gain personal influence and public recognition by frequent interactions with fans, or present desires and wishes that are not fulfilled in practical life to draw massive attention (Zhou et al., 2018). Despite their increasing influence in endorsements, online stars have not been consistently defined across scientific researches. The current study defines “online stars” as self-empowered individuals (Tiggemann and Slater, 2013) who gain fame and recognition by featuring self-generated content and frequently interacting with a large follower base on social media (Wood and Burkhalter, 2014; Schouten et al., 2020), becoming key opinion formers and online broadcasters and hence transforming social capital into cash.

Unlike online stars, social celebrities are often famous actors, singers, artists, supermodels, or athletes, etc., who gain social influence with their professional talent in specific fields and are frequently shown to general public *via* various media forms (Driessens, 2013). With their positive images in traditional media channels and social interactions, they gain fame and reputation and are greatly favored by certain social groups (Turner, 2004). Although online stars are sometimes also called “online/web celebrities,” the concept of “celebrities” is adopted in a more traditional sense in the current study, referring to individuals who become famous and popular by showing their personal talent and attractiveness, in conventional channels like movies, pictures, commercials and videos. As representatives of certain individual, social and commercial images, they are usually viewed by the public as cultural symbols.

Although celebrities and online stars share similarities, such as being considered to be popular, professional, and trustworthy (Choi and Rifon, 2007; Khamis et al., 2017), the two groups actually differ greatly. Social celebrities normally gain public recognition *via* traditional and social media, while online stars build their reputation almost exclusively by social media platforms. Further, unlike celebrities who gain fame through contents in various media forms, online stars become self-empowered with their self-generated contents. What’s more, online stars are often “grassroots” people from all walks of life (Russell and Rasolofoarison, 2017). Whereas traditional celebrities seem to be distant, online stars seem more like ordinary people and are perceived as closer to consumers. Hence, *social distance*, or the perceived psychological distance between the two types of endorsers and their fans, is the key factor differentiating celebrity endorsements and online star endorsements (Stever and Lawson, 2013; Liu and Qin, 2014).

### Celebrity Endorsements, Online Star Endorsements, and Consumers’ Purchase Intention

As the predictor of consumers’ buying behavior, purchase intention refers to the likelihood or desire of a customer to buy a certain product (Burke et al., 1985). Numerous factors affect purchase intentions, such as consumer personality characteristics, product internal cues, product external cues, and consumption scenarios, etc. (Dodds et al., 1991). Advertising, a combination of the above factors, has become an effective marketing tool. For both online and offline consumers, once they are exposed to different types of endorsement advertisements, they associate the content with the endorsed products, thereby increasing purchases intention.

Celebrity endorsement is a long-standing and effective driver of consumers’ purchase intention. Celebrities’ public image and fame engender trust in consumers and increase their willingness to purchase the endorsed products (Ayeh, 2015). Moreover, celebrities embody special and symbolic meanings in certain sociocultural environments. Thus, symbolic meaning is injected into the products or brands that they endorse *via* the process of meaning transfer in consumer purchasing (Knoll and Matthes, 2016). Consumers are also likely to buy products endorsed by celebrities due to the bandwagon and halo effects. In addition, research has demonstrated that advertisements with celebrity endorsements are associated with higher information processing speeds, better product evaluations and increased purchase intention, compared with advertisements lacking such endorsements (Rs and Alawadhi, 2020). Therefore, we propose the following hypothesis:

H1: Compared with no endorsement, celebrity endorsements have a positive effect on consumers’ purchase intention.

Online star endorsements represent a new type of endorsement. Similar to conventional celebrities in the sense that both are considered credible, popular and professional, online stars are recruited by more and more companies to present information and recommend purchases to their huge fan bases through impression management, expressiveness and persuasiveness. Online star endorsements are statements made by online celebrities in support of products or brands *via* advertisements on both traditional and social media. Based on the extant literature on famous persons, we posit that online star endorsements promote consumers’ purchase intention and that the attractiveness or personal charisma of these stars directly influences purchase intention (Park and Lin, 2020). More importantly, the unique qualities of online stars, such as their amiable, topical, and interactive nature, all contribute to consumers’ willingness to buy. Most online stars are considered “common” people and are naturally more amiable and appealing to consumers (Levordashka and Utz, 2016; Jin and Ryu, 2020). Therefore, consumers are more likely to accept online stars’ product recommendations. In public discussions, online stars are topical, and their comments boost interactions, thereby promoting the dissemination of their recommendations among the fans (Elmira and Oxana, 2018). Online stars also use social media frequently to communicate with fans, which further engenders trust from these fans, who unconsciously transfer this trust to the recommended product information (Gong and Li, 2017). Therefore, we propose the following hypothesis:

H2: Compared with no endorsement, online star endorsements have a positive effect on consumers’ purchase intention.

### Celebrity Endorsements, Online Star Endorsements, Ideal Self, and Actual Self

*Self-concept* is defined as a person’s cognitive and affective understanding of who and what they are (Joseph, 1982). Individuals have multiple selves but see themselves as a holistic self that is activated and stabilized (Wang et al., 2000). Following most literature on consumer research, we assume that consumers’ selves take the forms of an ideal self and an actual self (Sirgy, 1982). The ideal self is shaped by imagined ideals and goals related to what the person believes that he would like to be or aspire to become, whereas the actual self is based on the person’s perceived self-reality (Sirgy, 1982). When activated, self-concept determines one’s perception of and response to the environment. An individual is more willing to strengthen this self-concept and strive to seek self-enhancement or self-congruence through consuming behaviors, such as buying, presenting, or using certain products that are considered to represent an extension of the self. In this context, self-congruence theory postulates that consumers judge their self-image based on their perception of how closely their self-concept fits with the product’s image. Self-enhancement and self-congruence thus have been considered significant theories in analyses of the connection between consumer self-concept and purchase intention (Ahn et al., 2013).

According to *construal level theory*, people make abstract mental construals of distal events that differ by the dimensions of psychological distance. Specifically, as psychological distance increases, people construe objects at a higher (more abstract) level. High-level construals, which include the general, superordinate and core features of events, are simpler, more abstract, schematic, and decontextualized representations. In contrast, low-level construals, which include the incidental and subordinate features of events, are relatively more complex, concrete, contextualized, and unstructured representations. Four dimensions of psychological distance are described in construal level theory: temporal distance, social distance, spatial distance, and hypotheticality (Stephan et al., 2011). Despite important differences between these four dimensions, all of them affect the construal level similarly with respect to predicting different psychological states, as demonstrated in the literature (Milkman et al., 2010). Studies have found that, temporal distance triggers different construal levels for people, leading to the production of different selves (Kivetz and Tyler, 2007; Freitas et al., 2008; Rogers and Bazerman, 2008). The social distance generated by an individual’s perception of social objects should have a similar effect. Therefore, we introduce construal level theory to explain why celebrity endorsement and online star endorsement arouse different self-concepts.

When consumers view celebrity endorsements in advertisements, they focus on the celebrities, who are idols in their mind and are perceived to be socially distant. This triggers a high-level construal, motivating the consumer to represent themselves using abstract, core, essential and general features and generating a self-concept that congruent with their own or others’ expectations (Stephan et al., 2011). In this way, celebrity endorsements inspire the ideal self, and consumers strive to maintain a stable and coherent ideal self as a means of self-reflection. As the buying process is an interaction between advertised product information and consumer self-image (Knoll and Matthes, 2016), consumers are likely to change their attitude in response to the influence of celebrity endorsers. That is, activation of the ideal self leads consumers to pay attention to product self-presentation in advertisements. Congruence between product self-presentation and self-image drives consumers to associate the product with their personal image and social status, fostering a closer product–consumer connection (Su and Reynolds, 2017). Consumers who perceive the product as a projection of their self-image and a symbol of the endorsing celebrity buy it to maintain, extend and enhance their ideal selves (Hong and Zinkhan, 2010). Hence, the product is a better match for the ideal self and thus promotes purchase intention. Therefore, we propose the following hypothesis:

H3: The effect of celebrity endorsements on purchase intention is mediated by consumers’ ideal self.

Similarly, when consumers view online star endorsements in advertisements, they focus on the online stars, who are perceived to be common persons or even friends. This perception of natural social closeness results in a low-level construal, diverting consumer s’ attention from inner features. Thus, consumers represent themselves using concrete, superficial, subordinate and contextualized psychological features and thus generating a self-concept at close range (Qian and Park, 2021). In this way, online star endorsements activate the actual self, leading consumers to pay more attention to the actual functional value of the product, instead of its symbolic value. Strengthened as signifiers, online stars are more closely linked with the recommended products than with idols or dreams (Khamis et al., 2017). To speed up the spread of information, these stars usually aim to facilitate the understanding of product information and thus highlight a product’s functional value. In this case, consumers are likely to buy products endorsed by online stars to achieve a congruent self-image and extend the actual self (Sirgy, 1982). Therefore, we propose the following hypothesis:

H4: The effect of online star endorsements on purchase intention is mediated by the consumers’ actual self.

### Moderating Effects of Symbolic Appeal and Functional Appeal

Although social media platforms, such as Sino Weibo, WeChat, and Kuaishou, provide new opportunities for celebrities to promote themselves and interact with fans, the mysteriousness, distance, and idolatry associated with these celebrities remain unaffected. They remain symbolic as distant idols and dreams that are not readily approachable. The social distance between consumers and celebrities leads to activation of the ideal self, which represents consumers’ expectations and ambition. As a result, consumers who purchase the product are willing to trade some functional value, or even precious resources like time and money, in exchange for rewards, such as increased expression of their personality, identity and image. In advertisements, symbolic appeal means more emphasis on representing symbolic meaning for consumers, on the basis of creating an emotional state of mind for consumers (Johar and Sirgy, 1991; Choi et al., 2012). As symbolic appeal in advertising is anchored on creating ideal goals and images and delivering a certain lifestyle, social status, and signified meaning *via* products (Wolter et al., 2016), it is a better match for consumers whose ideal self is activated by celebrity endorsements. Such consumers are more likely to purchase products endorsed by celebrities to achieve a more congruent and better extended ideal self (Wang and Scheinbaum, 2018). Therefore, we propose the following hypothesis:

H5: Compared with functional appeal, symbolic appeal in advertisements strengthens the relationship between celebrity endorsements and consumers’ purchase intention.

Online stars use social media platforms to release pictures, texts and videos that represent real self-images and to interact frequently with their followers. As noted previously, online stars are perceived to be ordinary people, which reduces their social distance from consumers. These interactions are more likely to activate the actual self. To guide action, such consumers who purchase a product are more likely to sacrifice internal value for increased instrumental and practical value. Functional appeal in an advertisement involves informing consumers of the main functions, outstanding performance and comparative advantages of the product *via* concrete, peripheral, and functional messages (Johar and Sirgy, 1991; Ashley and Tuten, 2015). In this case, consumers’ actual self is triggered by online star endorsements, resulting in a preference for functional appeal in advertisements. Such consumers are more likely to purchase products endorsed by online stars to achieve a more congruent and better extended actual self (Ahn et al., 2013). Therefore, we propose the following hypothesis:

H6: Compared with symbolic appeal, functional appeal in advertisements strengthens the relationship between online star endorsements and consumers’ purchase intention.

## Methods and Results

### Study Overview

We conducted four studies to test our hypotheses. In Study 1, we examined the influences of celebrity and online star endorsements on consumers’ purchase intention and determined that both associations were positive. In Studies 2 and 3, we explored these positive associations and determined that they are mediated by consumers’ ideal self and actual self, respectively. Finally, in Study 4, we showed that this mediation effect is conditional upon the type of advertising appeals.

### Measurements

To measure the constructs in our studies, we used or adapted established scales with reference to prior research (see Table 1). A confirmatory factor analysis (CFA) including all study constructs produced a satisfactory overall fit. Factor loadings (more than 0.700), Cronbach’s alphas (over 0.700), CRs (greater than 0.700), and AVEs (ranging from 0. 529 to 0. 783) all point to high levels of reliability and convergent validity of the measurements (Table 1). In addition, the square root of the AVE value is higher than the correlation coefficients for all latent variables, indicating that high discriminant validity.

**TABLE 1:** Construct measurements.

Variables		Scale items	Factor loading	Cronbach’s α
Endorsers	Celebrities CR = 0.846 *AVE* = 0.578	A1: This celebrity interacts with fans occasionally, keeping a sense of mystery.	0.744	0.715
		A2: This celebrity is viewed as an idol, with a strong sense of distance.	0.755	
		A3: This celebrity has personal, public, social, and commercial images.	0.767	
		A4: This celebrity is authoritative.	0.774	

	Online stars *CR* = 0.892 *AVE* = 0.673	B1: This online star interacts with fans frequently and is amiable.	0.834	0.737
		B2: This online star is viewed as a friend, with little sense of distance.	0.833	
		B3: This online star has personal and commercial images.	0.801	
		B4: This online star is very down to earth.	0.813	

Consumers’ self-concept	Ideal self *CR* = 0.882 *AVE* = 0.713	C1: I can feel that the scenario describes my ideal self-image.	0.879	0.835
		C2: I can feel that the scenario reflects my perfect self-state.	0.853	
		C3: Through the scenario, I can positively associate with the ideal self.	0.800	

	Actual self *CR* = 0.901 *AVE* = 0.752	D1: I can feel that the scenario describes my real self-image.	0.902	0.878
		D2: I can feel that the scenario corresponds to my real self-state.	0.864	
		D3: Through the scenario, I can objectively associate with the real self in my work.	0.834	

Advertising Appeals	Symbolic appeal *CR* = 0.908 *AVE* = 0.766	E1: The product recommended in the advertisement is for people who want the best things in life.	0.856	0.846
		E2: The product recommended in the advertisement makes me stand out in a crowd.	0.889	
		E3: The product recommended in the advertisement promotes my self-image.	0.881	

	Functional appeal *CR* = 0.915 *AVE* = 0.783	F1: The product recommended in the advertisement is for people who want the most practical things in life.	0.868	0.861
		F2: The product recommended in the advertisement brings conveniences in my life.	0.909	
		F3: The product recommended in the advertisement is very useful to me.	0.877	

Consumers’ purchase intention *CR* = 0.818 *AVE* = 0.529		G1: I am willing to know more about this product.	0.755	0.721
		G2: I am willing to save the product information for future purchasing reference.	0.738	
		G3: I will like the product recommended in this advertisement.	0.700	
		G4: I am willing to share, discuss and recommend this product with others.	0.716	

### Pilot Study and Stimuli

We conducted a pilot study to determine the appropriate stimuli for formal experiments. To eliminate confounding variables and ensure good validity and reliability, we used real celebrities and online stars as the experimental dataset. We also drew on authoritative selection methods developed by scholars, such as Sengupta et al. (1997) and Biswas et al. (2006) to ensure that suitable stimuli were selected for this study.

First, we selected all celebrities who appeared in all rankings, such as Baidu Billboard, Tencent Billboard, and Weibo Billboard, ranked them in order, and finally identified 10 celebrities of different genders, excluding those who had received negative publicity. Second, using the same selection method, we listed 20 online stars who appeared in all rankings, such as China online stars big data report, listed them in general order and finally selected 10 online stars of different genders. Third, we invited 55 undergraduates (M_*age*_ = 21.257 years; 30 female) at a comprehensive university in Wuhan to complete a questionnaire. In this questionnaire, we listed the names of the 10 stars and 10 online stars and attached their photos, which were modified using Photoshop to ensure that they were identical in size, brightness, pixels, and colors. To rule out effects of popularity, expertise, trustworthiness and attractiveness, we introduced the widely used celebrity trait scale developed by Ohanian (1991) and Ding et al. (2005). Specifically, subjects rated on the popularity, expertise, trustworthiness and attractiveness of the celebrities and online stars on 7-point semantic differential scales. For example, expertise was measured by four items with 7-point scales: expert/not an expert, experienced/inexperienced, qualified/unqualified, skilled/unskilled.

Using data from 52 valid questionnaires (those that lacked key information or were answered sloppily were ruled out), we selected Zhao Liying and Papi Jiang, who received the highest scores, to represent female celebrities and online stars, respectively. As shown in Table 2, we found no significant difference between the respondents’ perceptions of these stimuli in terms of popularity, expertise, trustworthiness, and attractiveness. We further selected Yang Yang and Liu Yu, who also received the highest scores, to represent male celebrities and online stars, respectively. Similarly, we found no significant differences between the respondents’ perception of the common features of the celebrity and online star (Table 2). To better allow the participants to distinguish between celebrities and online stars in the following formal experiments, we added a text description of each to the stimulus materials, drawing on the research methods of Atkin and Block (1983).

**TABLE 2:** Mean scores of the properties of celebrities and online stars.

	Popularity			Expertise			Trustworthiness			Attractiveness		
	Celebrities	Online stars	*P*	Celebrities	Online stars	*P*	Celebrities	Online stars	*P*	Celebrities	Online stars	*P*
Pilot study	4.804	4.644	0.662	4.550	4.588	0.870	4.921	4.867	0.873	5.279	5.078	0.555
Women	1.375	1.083		0.572	0.855		1.096	1.133		1.150	1.188	
Pilot study	4.737	4.933	0.432	4.618	4.529	0.655	4.983	5.047	0.857	5.035	4.800	0.417
Men	1.075	0.740		0.749	0.769		1.327	1.232		0.777	1.112	
Study 1	4.688	4.744	0.807	4.564	4.532	0.843	4.462	4.403	0.830	5.302	5.032	0.340
	1.011	0.937		0.795	0.464		1.115	1.205		1.009	1.309	
Study 2	4.702	4.667	0.705	4.533	4.426	0.671	4.521	4.596	0.640	5.143	5.227	0.617
	0.961	0.973		1.103	1.156		0.501	0.830		1.017	1.307	
Study 3	4.516	4.685	0.113	4.346	4.375	0.872	4.865	4.635	0.115	5.004	5.122	0.470
	0.452	0.859		1.154	1.243		0.960	0.984		0.938	1.188	
Study 4	4.662	4.836	0.150	4.396	4.455	0.647	4.523	4.571	0.570	5.099	4.893	0.120
	1.007	0.949		1.134	1.149		0.781	0.818		0.975	1.148	

***p < 0.01, **p < 0.05, *p < 0.1.

After setting our research stimuli, we processed photos of the celebrities, online stars and products as described earlier, and used Photoshop to combine these photos into single images. In each image, the celebrity or online star is shown on the left side and the product is shown in a relatively smaller shape on the right side.

### Study 1

In Study 1, we explored the effects of celebrity endorsements and online star endorsements on consumers’ purchase intention. We selected a facial cleanser endorsed both by celebrities and by online stars, using a fictitious brand X to rule out any effect of brand association.

#### Participants and Procedure

One hundred and twenty participants (recruited both on-campus and off-campus) were invited to complete a questionnaire in exchange for a bookmark of ¥2 as a gift. 117 valid questionnaires were collected (M_*age*_ = 22.836 years, SD_*age*_ = 4.472 years; 56.9% female). Three questionnaires were considered invalid because they lacked key information or were answered sloppily.

**FIGURE 1:**
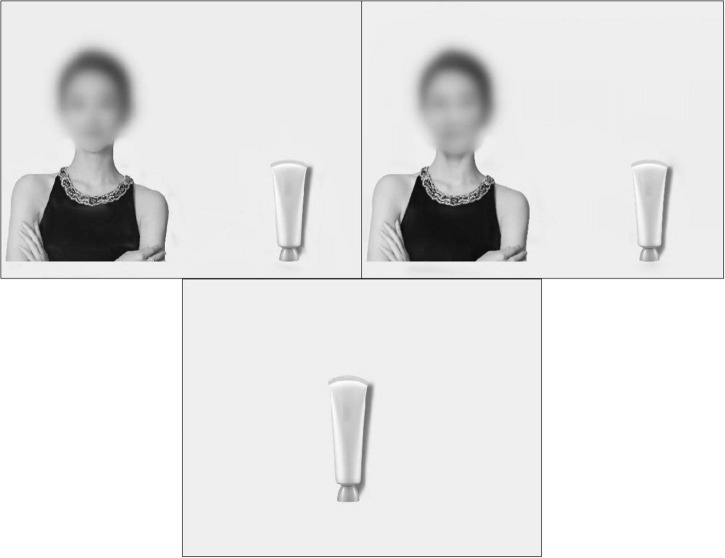
Stimulus material in Study 1 (blurred for legal and copyright reasons). On a famous online shopping site, an advertisement for Brand X facial cleanser is endorsed by celebrities (is endorsed by online stars/is not endorsed). Image reproduced with permission from Rui Chu Cultural Development Co., Ltd.

First, participants were randomly assigned to one of three conditions: celebrity endorsement, online star endorsement or no endorsement (shown in Figure 1). They were given materials describing certain conditions, as in the following example: “You want to buy a facial cleanser. When you are browsing information on a famous online shopping site, you see an advertisement for Brand X facial cleanser endorsed by celebrities [endorsed by online stars/that is not endorsed].” We described celebrities and online stars in the stimulus material and selected Zhao Liying and Papi Jiang as examples of celebrities and online stars, respectively. Specifically, in the celebrity endorsement materials we described as follows: “Zhao Liying is now a top star, known as the most popular actress.” In the online star endorsement materials, the description read “Papi Jiang is an online star with a large number of fans, who is rated as the most popular online personality.” Then, referring to the pilot study, we measured the common features of them, to rule out the effects of these characteristics. After that, the participants were asked to complete the remaining three parts of the questionnaire. In the first part, we presented advertisements featuring celebrities, online stars or no endorsers to the participants, who were then asked to rate items concerning obvious celebrity and online star characteristics, such as “This celebrity is viewed as an idol, with a strong sense of distance” (Table 1). The items were adapted from scales by Hess (2003); Stever and Lawson (2013), and Cohen et al. (2016). In the second part, participants were asked to evaluate their purchase intention to purchase the product under the three conditions. This section was based on the scale by Dodds et al. (1991) and Ma et al. (2015), with four items, such as “I will like the product recommended in this advertisement” (Table 1). All items in this study were rated on 7-point Likert scales (1 = “strongly disagree,” 7 = “strongly agree”). In the last part, participants entered their demographic information.

#### Results

##### Manipulation Check

We manipulated the perceptions of celebrity endorsements and online star endorsements by assigning participants to different conditions. The results revealed that participants’ perceptions of the endorsing conditions were consistent with the types of endorsers. A significantly greater number of participants in the celebrity endorsement condition considered the advertisement as being endorsed by celebrities (M_*celebrity endorsement*_ = 5.129, *SD* = 1.017; M_*online star endorsement*_ = 3.397, *SD* = 0.747, *p* < 0.001). In the condition of online star endorsement, a significantly greater number of participants considered the advertisement as being endorsed by online stars (M_*celebrity endorsement*_ = 2.939, *SD* = 0.887; M_*online star endorsement*_ = 4.871, *SD* = 0.687, *p* < 0.001). Therefore, the manipulation of celebrities and online stars was effective. As shown in Table 2, there were no significant differences between the participants’ perception of the common traits of celebrities and online stars (M_*celebrity popularity*_ = 4.688, *SD* = 1.011; M_*online star* popularity_ = 4.744, *SD* = 0.937, *p* = 0.807), (M_*celebrity expertise*_ = 4.564, *SD* = 0.795; M_*online star* expert*ise*_ = 4.532, *SD* = 0.464, *p* = 0.843), (M_*celebrity trustworthiness*_ = 4.462, *SD* = 1.115; M_*online star* trustworthiness_ = 4.403, *SD* = 1.205, *p* = 0.830), (M_*celebrity attractiveness*_ = 5.302, *SD* = 1.009; M_*online star attractiveness*_ = 5.032, *SD* = 1.309, *p* = 0.340).

##### Hypothesis Testing

One-way ANOVA showed that differences in the product endorsers influenced participants’ willingness to purchase products [*F*(1, 117) = 37.135, *p* < 0.001]. Compared with no endorsement, both celebrity endorsement (M_*celebrity endorsement*_ = 4.903, *SD* = 0.784; M_*control*_ = 3.613, *SD* = 0.656, *p* < 0.001) and online star endorsement increased participants’ purchase intention (M_*online star endorsement*_ = 4.772, *SD* = 0.766; M_*control*_ = 3.613, *SD* = 0.656, *p* < 0.001). These results support Hypotheses 1 and 2. The results also showed that the influences of celebrity endorsers and online star endorsers on consumers’ purchase intention were not significantly different.

#### Discussion

The results of Study 1 mainly verified the effects of celebrity and online star endorsements on consumers’ purchase intention and proved a lack of significant difference between these effects. However, the factors driving this effect were not examined. Therefore, we conducted the following study to explore the mediating mechanism.

### Study 2

The main purpose of Study 2 was to verify that celebrity and online star endorsements activate consumers’ ideal self or actual self, respectively, thereby enhancing their purchase intention. Specific stimulus information for study 2 is available in Supplementary Appendix.

#### Participants and Procedure

One hundred and fifty participants (recruited both on-campus and off-campus) were asked to complete the questionnaire in exchange for a bookmark of ¥2 as a gift. 137 valid questionnaires were collected (M_*age*_ = 23.456 years, SD_*age*_ = 4.451 years; 58.1% female). 13 questionnaires were ruled out because they lacked key information or were answered sloppily.

Like in Study 1, we selected Zhao Liying and Papi Jiang as endorsers in this study; however, we replaced the facial cleanser with a computer, which was fictitiously branded “X” to ensure the effectiveness of manipulation by avoiding associations with existing brands. We replicated the manipulation procedure involving all other pictures used in Study 1.

We also conducted an experiment to examine the psychological mechanism by which celebrity endorsements and online star endorsements influence purchasing behavior. We replicated all the materials (except the product) and steps used in the previous study and added a new step in which the participants were asked to score their ideal and actual selves using a 4-item scale developed by Cohen et al. (2016). This step was taken after completion of the first two steps of the experiment. As shown in Table 1, the constructs of ideal self and actual self were each measured by three items on 7-point Likert scales.

#### Results

##### Manipulation Check

We first confirmed the effectiveness of all manipulations. The results revealed that a significantly greater number of participants in the celebrity endorsement condition considered the advertisement as being endorsed by traditional celebrities (M_*celebrity endorsement*_ = 4.889, *SD* = 0.682; M_*online star endorsement*_ = 2.924, *SD* = 0.782, *p* < 0.001). A significantly greater number of participants in the online star endorsement advertisement condition considered the advertisement as being endorsed by online stars (M_*online star endorsement*_ = 5.095, *SD* = 1.002; M_*celebrity endorsement*_ = 3.552, *SD* = 0.852, *p* < 0.001). Therefore, the manipulation of celebrities and online stars was effective. There were no significant differences in the participants’ perceptions of common traits between the celebrities and online stars (M_*celebrity popularity*_ = 4.702, *SD* = 0.961; M_*online star* popularity_ = 4.667, *SD* = 0.973, *p* = 0.705), (M_*celebrity expertise*_ = 4.533, *SD* = 1.103; M_*online star* expert*ise*_ = 4.426, *SD* = 1.156, *p* = 0.671), (M_*celebrity trustworthiness*_ = 4.521, *SD* = 0.501; M_*online star* trustworthiness_ = 4.596, *SD* = 0.830, *p* = 0.640), (M_*celebrity attractiveness*_ = 5.143, *SD* = 1.017; M_*online star* attractiveness_ = 5.277, *SD* = 1.307, *p* = 0.617).

##### Hypothesis Testing

We conducted a one-way ANOVA to show that different product endorsers influenced the participants’ willingness to purchase the product [*F*(1, 137) = 39.753, *p* < 0.001]. Compared with no endorsement, both celebrity endorsement (M_*celebrity endorsements*_ = 4.893, *SD* = 0.748; M_*control*_ = 3.694, *SD* = 0.664, *p* < 0.001) and online star endorsement led to increases in purchase intention (M_*online star endorsement*_ = 4.760, *SD* = 0.767; M_*control*_ = 3.694, *SD* = 0.664, *p* < 0.001). In different endorsing conditions, we found no significant difference in consumers’ purchase intention when the product was changed.

##### Mediation Analysis

**FIGURE 2:**
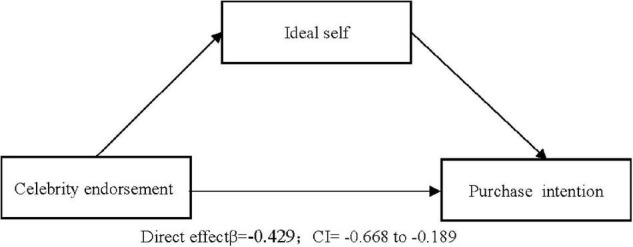
Mediating analysis of celebrity endorsement on purchase intention.

**FIGURE 3:**
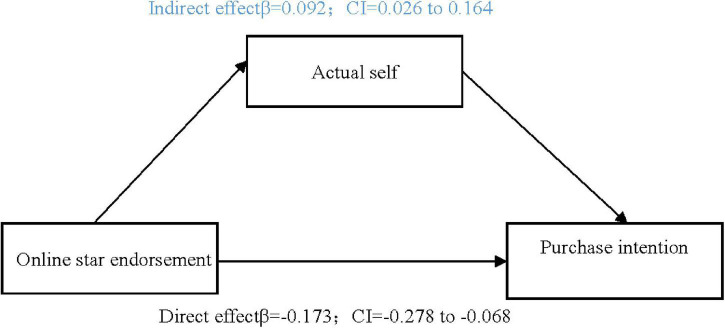
Mediating analysis of online star endorsement on purchase intention.

Bootstrap analysis was adopted in the current research to test mediation, since causal step regression is limited to models with one mediation variable and continuous variables. As the main effect was verified in Study 1 and 2, referring to Schouten et al. (2020)’s research process, we only reported the mediating effect results. With a sample size of 5,000 and 95% confidence interval (CI), the mediating effect of ideal self on celebrity endorsement was significant (Figure 2), as the CI did not include 0 (BootLLCL = −0.239, BootULC = -0.090). In contrast, the mediating effect of actual self under this condition was not significant, as the CI contained 0 (BootLLC = −0.076, BootULCL = 0.019). Meanwhile, the mediating effect of actual self on online star endorsement was significant (Figure 3), as the CI did not include 0 (BootLLCL = 0.026, BootULCL = 0.164), whereas the mediating effect of ideal self under this condition was not significant, with a CI containing 0 (BootLLC = −0.011, BootULC = 0.074). In summary, celebrity endorsements and online star endorsements led to activation of the participants’ ideal self and actual self, respectively, supporting Hypotheses 3 and 4.

#### Discussion

In addition to providing further support for Hypotheses 1 and 2, the results of Study 2 also provide support for Hypotheses 3 and 4, as they verified the mediating role of self-concept in the effects of celebrity endorsements and online star endorsements on purchase intention. To further explore the mediating role of self-concept from a different perspective, we conducted Study 3 using a priming approach. In the following section, we describe the selection of male rather than female celebrities and online stars and further alteration of product types.

### Study 3

In this study, we followed the approach developed by Kettle and Häubl (2011) to activate the ideal and actual selves, by using the situation of drinking beer in a public or private location as the stimulus (Graeff, 1997), which is an established way to activate self-concept.

We proved in Study 2 that celebrity endorsements activate consumers’ ideal self, whereas online star endorsements activate the actual self, both of which promote purchase intention. We then assume that, exposed to celebrity endorsements, consumers with activated ideal self are more willing to buy products endorsed by celebrities. Similarly, after exposure to online star endorsements, consumers with activated actual self are more likely to find online star-endorsing products attractive. To further test our assumptions, we conducted Study 3.

#### Participants and Procedure

Two hundred participants (recruited both on-campus and off-campus) took part in the study in exchange for a bookmark of ¥2 as a gift. 183 valid questionnaires were collected (M_*age*_ = 23.803 years, SD_*age*_ = 5.173 years; 59.6% female). Seventeen questionnaires were ruled out because they lacked key information.

In Study 3, we replaced female endorsers with males, specifically Yang Yang and Liu Yu, as selected in the pilot study. Specific stimulus information for study 3 is available in Supplementary Appendix. Similar to study 1, study 3 included a description of male celebrities and online stars. The male celebrity is described as follows, “Yang Yang is a celebrity with a large number of fans and is the most popular celebrity.” As for online stars, participants read “Liu Yu, an online star with a large number of fans, is viewed as a popular online star.” We also replaced the computer used in Study 2 with shampoo, which was branded fictitiously as “X.” We used a 2 (ideal self vs. actual self) × 3 (celebrity vs. online star vs. control) between-subjects design to further test the mediation effect. First, based on the experimental method described by Graeff (1997) to activate ideal self and actual self, we assigned participants randomly to either the ideal self or the actual self group and exposed them to materials that encouraged activation of the ideal or the real self-concept, respectively. The material for ideal self condition read as follows, “Imagine you and your friends in a restaurant, bar, lounge, or other public places. You are considering to buy a dozen of beer and have decided to drink them in the public. That is to say, when you drink the beer, there will be a lot of people watching you and the way you drink beer.” For actual self condition, it read “Suppose you are considering to buy a dozen of beer, and then go home to drink them to relax. Namely, you will drink beer in a private place with no one else present (excluding family members). At this time, you can drink while watching a rented movie, your favorite TV shows, or sports events on TV.” Next, we asked the participants to complete the ideal and actual self scales and report their intention to buy the product. Similarly, we measured the popularity, expertise, trustworthiness, and attractiveness of both celebrities and online stars to rule out the effects of these factors.

#### Results

##### Manipulation Check

We first confirmed the effectiveness of our manipulation. The results of our check revealed that the participants in the actual self group indeed perceived a more realistic version of themselves than those in the ideal self group (M_*actual self*_ = 5.019, *SD* = 0.886; M_*ideal self*_ = 3.500, *SD* = 0.863, *p* < 0.001). The participants in the ideal self group reported a higher ideal vs. actual self score (M_*ideal self*_ = 5.008, *SD* = 0.672; M_*actual self*_ = 3.119, *SD* = 0.922, *p* < 0.001). We further determined that the manipulation of celebrity endorsements and online star endorsements is appropriate. Our results showed that participants in celebrity endorsement group indeed perceived the advertisement as being endorsed by a celebrity, scoring higher than online star endorsements (M_*celebrity endorsement*_ = 4.961, *SD* = 0.671; M_*online star endorsement*_ = 3.377, *SD* = 0.953, *p* < 0.001), whereas in online star endorsement group, participants reported significantly higher scores for online star endorsements than celebrity endorsements (M_*online star endorsement*_ = 5.128, *SD* = 0.700; M_*celebrity endorsement*_ = 3.190, *SD* = 0.765, *p* < 0.001). In addition, no significant difference was observed between the participants’ perceptions of the common properties of celebrities and online stars (shown in Table 2).

##### Mediating Effect Analysis

**FIGURE 4:**
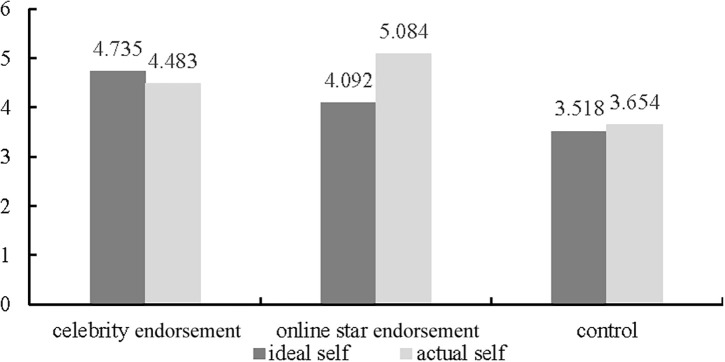
Effects of endorsement types and self-concept on purchase intention.

A one-way ANOVA revealed a significant interaction between consumer self-concept and endorsement type [*F*(1, 183) = 12.184, *p* < 0.001]. Specifically, participants with activated ideal self-concept are more likely to be motivated to purchase the product with celebrity endorsements compared with those in the online star endorsement group and control group (M_*celebrity endorsement*_ = 4.735, *SD* = 0.673; M_*online star endorsement*_ = 4.092, *SD* = 0.835; M_*control*_ = 3.518, *SD* = 0.909, *p* < 0.001) (Figure 4). However, when actual self was activated, participants in the online star endorsement group had a higher purchase intention than those in the celebrity endorsement group and control group (M_*online star endorsement*_ = 5.084, *SD* = 0.742; M_*celebrity endorsement*_ = 4.483, *SD* = 0.571; M_*control*_ = 3.654, *SD* = 0.738, *p* < 0.001) (Figure 4). That is, online star endorsements increased purchase intention by activating consumers’ actual self-concept. The results supported Hypotheses 3 and 4.

#### Discussion

The results of Study 3 further proved that consumers’ self-concept is a key factor connecting celebrity and online star endorsements with consumers’ purchase intention. However, the first three experiments did not examine the boundary conditions of these effects. We conducted Study 4 to test the moderating effect of appeal types in advertisement.

### Study 4

In this study, to examine the conditional factors for the effects of celebrity endorsements and online star endorsements on purchase intention, we introduced symbolic appeal and functional appeal in advertisements as moderating variables. We then sought appropriate types of verbal descriptions in advertisements. We conducted this study to test Hypotheses 5 and 6.

#### Participants and Procedure

Three hundred adults (recruited both on-campus and off-campus) participated in exchange for a bookmark of ¥2 as a gift. 262 valid questionnaires were collected (M_*age*_ = 23.145 years, SD_*age*_ = 4.663 years; 39.3% female). Thirty-eight incomplete questionnaires were ruled out.

We selected celebrities and online stars using the same process described in Study 1, replacing facial cleanser used in that study with a mobile phone, which we branded fictitiously as “Y” to ensure the effectiveness of manipulation.

The experiment was most similar to that of Study 1, except that the moderating variables were included and measured. We employed a 2 (advertising appeal: symbolic vs. functional) × 3 (endorser: celebrity vs. online star vs. control) between-subjects design to further test the moderating effect. First, the participants were randomly assigned to four treatment groups and two control groups. They read materials similar to those used in Study 1, only with a different product type. Specific stimulus information for this study is available in Supplementary Appendix. Next, we manipulated the advertisement appeal by presenting stimulus materials to the participants and describing the symbolic or functional value accordingly. The symbolic appeal description was as follows: “This mobile phone makes you more attractive, helps expressing your personality fully in the crowd, creates a perfect image and ultimate experience for you.” For functional appeal, the description read: “This mobile phone is absolutely easy to use, with fast operating speed, long battery life, short charging time and ultra-wide camera angle, meeting various needs for work and entertainment.” We also measured popularity, expertise, trustworthiness, and attractiveness of both celebrities and online stars to rule out the effects of these characteristics. Then, we exposed the participants to advertisements endorsed by either celebrities or online stars, or with no endorsers and asked to them complete the measurement items. They also rated the materials in terms of symbolic appeal and functional appeal based on a scale developed by Bhat and Reddy (1998). As shown in Table 1, symbolic appeal and functional appeal were each measured with three items on 7-point Likert scales (1 = strongly disagree; 7 = strongly agree), such as “The products recommended in this advertisement are for people who want the best things in life.”

#### Results

##### Manipulation Check

A one-way ANOVA was used to confirmed the effectiveness of our manipulation. As expected, participants in the celebrity endorsement group reported significantly higher scores for celebrity endorsements than for online star endorsements (M_*celebrity endorsement*_ = 5.221, *SD* = 0.845; M_*online star endorsement*_ = 3.546, *SD* = 0.769, *p* < 0.001). Participants in the online star endorsement group reported significantly higher scores for online star endorsements than for celebrity endorsement (M_*online star endorsement*_ = 5.039, *SD* = 0.715; M_*celebrity endorsement*_ = 3.071, *SD* = 0.852, *p* < 0.001).

In symbolic appeal group, participants assigned significantly higher scores to symbolic appeal than to functional appeal (M_*symbolic appeal*_ = 5.118, *SD* = 0.666; M_*functional appeal*_ = 3.541, *SD* = 0.982, *p* < 0.001). Conversely, in functional appeal group, participants assigned significantly higher scores to functional appeal than to symbolic appeal (M_*functional appeal*_ = 5.181, *SD* = 0.707; M_*symbolic appeal*_ = 3.216, *SD* = 0.724, *p* < 0.001). Further, as shown in Table 2, participants did not perceive significant differences between the common traits of celebrities and online stars.

##### Moderating Effect Analysis

**FIGURE 5:**
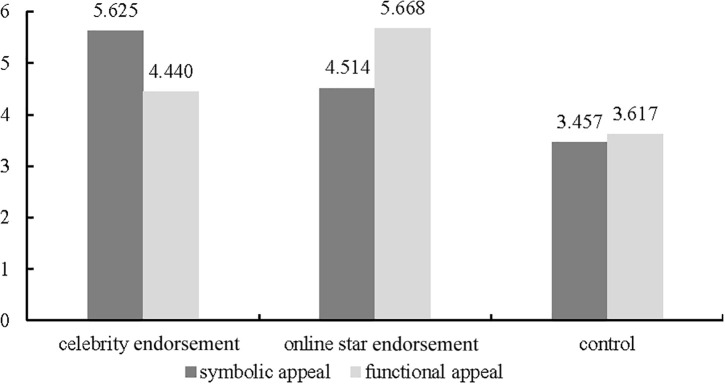
Effects of endorsement types and advertising appeals on purchase intention.

The results of the interaction test showed a significant moderating effect [*F*(1, 262) = 19.328, *p* < 0.001]. Specifically, in the symbolic appeal condition, celebrity endorsements had a significantly stronger effect on purchase intention (M_*celebrity endorsement*_ = 5.625, *SD* = 0.450) than online star endorsements (M_*online star endorsements*_ = 4.514, *SD* = 0.558) and no endorsement (M_*control*_ = 3.457, *SD* = 0.834) (*p* < 0.001) (Figure 5). In the functional appeal condition, online star endorsement had a significantly stronger effect on purchase intention (M_*online star endorsement*_ = 5.668, *SD* = 0.453) than celebrity endorsement (M_*celebrity endorsement*_ = 4.440, *SD* = 0.498) and no endorsement (M_*control*_ = 3.617, *SD* = 0.705) (*p* < 0.001) (Figure 5). In other words, symbolic appeal is more closely matched with celebrity endorsements as a driver of consumers’ purchase intention, while functional appeal is more closely matched with online star endorsements, thus supporting Hypotheses 5 and 6.

#### Discussion

Following the first three studies, we introduced advertising appeal as a moderating variable in Study 4. Our results revealed that the mechanism underlying the influences of different types of endorsement on consumers’ purchase intention differs for advertisements involving symbolic appeal vs. functional appeal.

## General Discussion

Research on celebrity endorsement generally suggest that popularity, expertise and trustworthiness increase consumers’ purchase intention (Carroll and Ahuvia, 2006; Wang and Scheinbaum, 2018). However, few studies have focused on the effects of celebrity endorsements and online star endorsements on purchase intention (Jin and Ryu, 2020; Schouten et al., 2020). We hypothesized that self-concept and advertisement appeal play significant roles in shaping consumers’ purchase intention. Across four studies, we confirmed the following: (1) Both celebrity endorsements and online star endorsements increase consumer purchase intention, in ways that are not significantly different. Similar to traditional celebrities, online stars also attract and convince consumers using their popularity, trustworthiness and expertise (Zamudio, 2016). Furthermore, online stars interact more frequently with consumers and are therefore considered more like friends, enhancing consumers’ reliance on and trust in online star endorsements and drives purchase intention. (2) Self-concept plays a mediating role in this process. Consumers whose ideal self-concept is activated by celebrities are more willing to buy products endorsed by celebrities, who are perceived to be socially distant. However, consumers whose actual self-concept is activated by online stars are more likely to purchase products endorsed by them. (3) Matching between different advertising appeals and endorsement types relates to the effects of endorsements on consumers’ purchase intention. When ideal self is activated, consumers seek a product reward related to personality or identity-expression. As a symbolic appeal in an advertisement conveys social status and meaning of the product, consumers’ willingness to buy increases when this appeal is matched with a celebrity endorsement. Similarly, consumers’ purchase intention is higher when a functional appeal is matched with online star endorsements because consumers seek instrumental and utilitarian product rewards once their actual self has been activated.

### Theoretical Contributions

The current research is among the first to precisely define the concept of online stars and differentiate it from the concept of celebrities in a traditional sense. As most prior studies focus on the effects of celebrity endorsements (Atkin and Block, 1983; Biswas et al., 2006), the influence of online star endorsements is rather understudied due to limited differentiation between the two groups. Though online stars are distinct from celebrities in the sense that they are socially much closer to consumers, our research suggests that celebrity and online star endorsements are not significantly different in their ability to increase consumers’ purchase intention. Seemingly incongruent with Schouten et al. (2020)’s findings, where significant difference in trustworthiness was found between online stars and celebrities, our study shows that both groups influence purchase intention likewise, as we understand online stars as similarly trustworthy to traditional celebrities since they are both famous to the public.

A second contribution is the importance of exploring the mechanism underlying the effects of online star endorsements and celebrity endorsements. Based on the theories of self and construal level theory, our results show that self-concept mediates the influence of online star endorsements and celebrity endorsements on purchase intention. While extant literature on endorsement effects seem rather confined to investigating the expertise, popularity, trustworthiness, and attractiveness of the endorsers (Park and Lin, 2020; Schouten et al., 2020), our research provides a new perspective by examining consumers’ self-concept in different endorsing situations, supplementing the literature on self-concept and consumer purchase intention (Oumlil and Erdem, 1997).

Third, instead of considering emotional/rational appeal as boundary conditions like previous endorsement studies did (Ruiz and Sicilia, 2004; Andreu et al., 2015), we have found a better moderator using symbolic/functional appeal when investigating celebrity/online star endorsements effects. By examining the moderating effect of advertising appeals, we have gained a better understanding of the situations wherein celebrity endorsements and online star endorsements increase purchase intention.

### Practical Implications

Our research has important practical implications for advertising and product marketing researches. When considering endorsers, managers may select both celebrities and online stars in accordance with current marketing practice (Milkman et al., 2010), as they influence purchase intention in a similarly significant way. For example, Mercedes-Benz selected traditional celebrities and a popular streamer to endorse its products.

Second, our results also suggest managers consider the significance of appropriately matching the advertising appeal with the endorser type. Specifically, companies may adopt symbolic appeal when a celebrity endorses their product, reinforcing consumers’ ideal self and promoting purchase intention. Similarly, using functional appeal to match online star endorsers, companies activate consumers’ actual self and drive them to buy.

Third, enterprises should consider promoting purchase intention by activating consumer self-concept, which is dynamic and malleable (Freitas et al., 2008; Hong and Zinkhan, 2010). Different activation of consumers’ self-concept increases their willingness to buy products endorsed by either celebrities or online stars to maintain a congruent self. Therefore, celebrity-endorsed products might describe themselves for benefits, such as personal charisma or perfect image in public places, to activate the ideal self. Likewise, companies using online star endorsements to activate the actual self, might encourage consumers to purchase by providing information on product core functions to meet their diverse needs.

### Limitations and Future Research

This research also has some limitations. First, research on online stars remains at a nascent stage. Although we included celebrities and online stars of both sexes in our studies, we failed to include online stars of an older age because most of them are between 18 and 35. Future research might examine the influence of celebrity endorsers and online star endorsers of different ages on consumers’ purchase intention as the pool of online stars expands. Second, this research only validates the interaction of advertising appeals with endorsement types. Future research could examine other possible moderating variables, such as consumer cultural factors, consumer involvement, communication channels and consumption contexts. Third, this research only measures the independent effects of celebrity or online star endorsers. Future studies could investigate the influence of using a combination of celebrity and online star endorsements on consumers’ purchase intention.

## Data Availability Statement

The raw data supporting the conclusions of this article will be made available by the authors, without undue reservation.

## Ethics Statement

All procedures performed in studies involving human participants were reviewed and approved by the School of Economics and Management, Xinan University, China. The participants gave written informed consent to participate in this study.

## Author Contributions

PS was involved in the whole process of the research. XL, CS, LW, and BG contributed to conduct the experiments and perform the statistical analysis. PS, XL, and YZ wrote, revised, read the manuscript, and reviewed and coordinated the final article as a whole. All authors contributed to the article and approved the submitted version.

## Conflict of Interest

The authors declare that the research was conducted in the absence of any commercial or financial relationships that could be construed as a potential conflict of interest.

## Publisher’s Note

All claims expressed in this article are solely those of the authors and do not necessarily represent those of their affiliated organizations, or those of the publisher, the editors and the reviewers. Any product that may be evaluated in this article, or claim that may be made by its manufacturer, is not guaranteed or endorsed by the publisher.
