# Supplementary material for: Online Star vs. Celebrity Endorsements: The Role of Self-Concept and Advertising Appeal in Influencing Purchase Intention
Source: Front Psychol. 2021 Nov 23;12:736883. doi: 10.3389/fpsyg.2021.736883 (PMC8650705; doi:10.3389/fpsyg.2021.736883)

Appendix. Stimuli used in study 1-4. (Pictures burred for legal and copyright reasons)

Note: In actual experiments, no photos of celebrities and online stars were blurred, so as to distinguish between the two types of endorsers. Images reproduced with permission from Rui Chu Cultural Development Co., Ltd.

Study 1

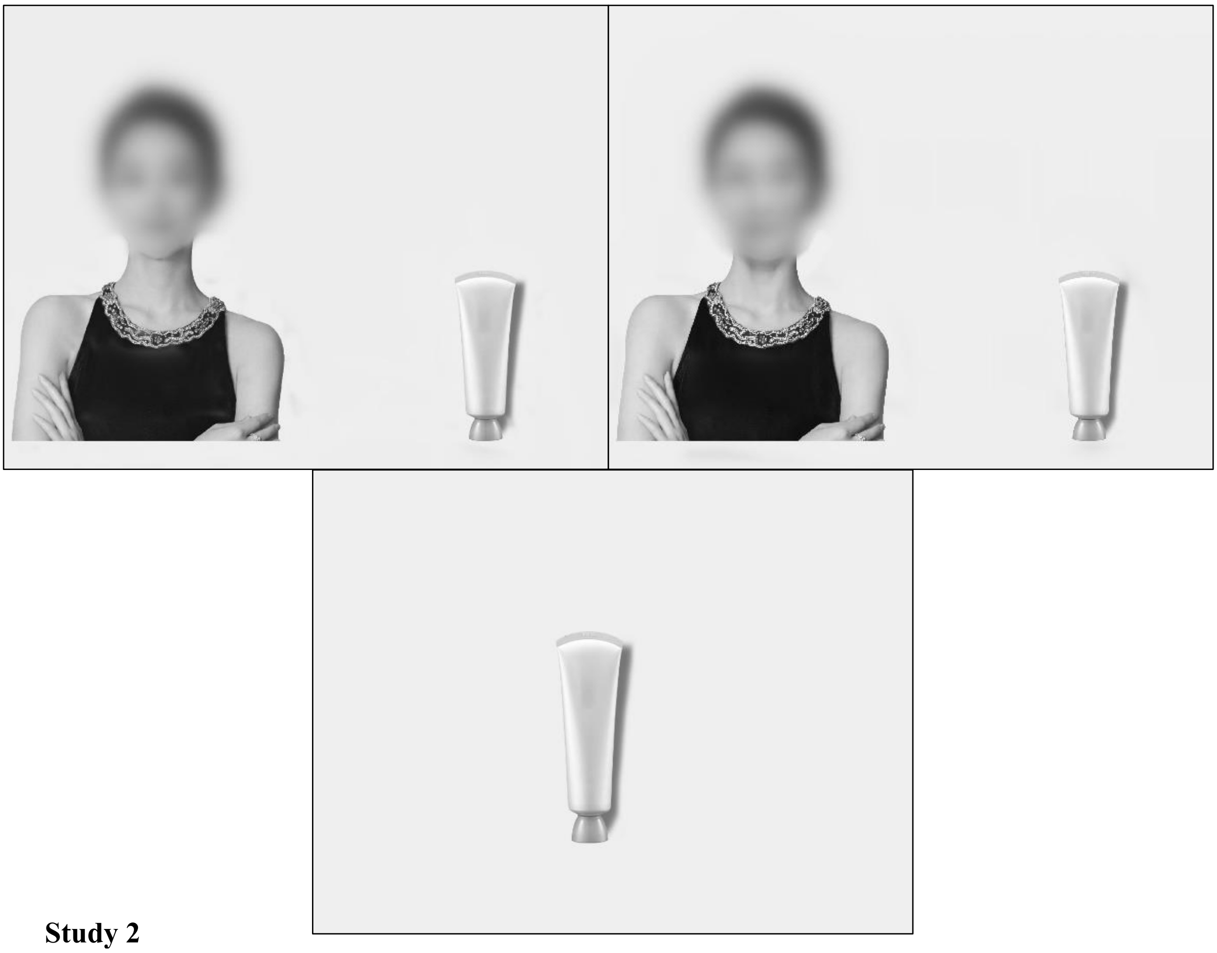

Study 2

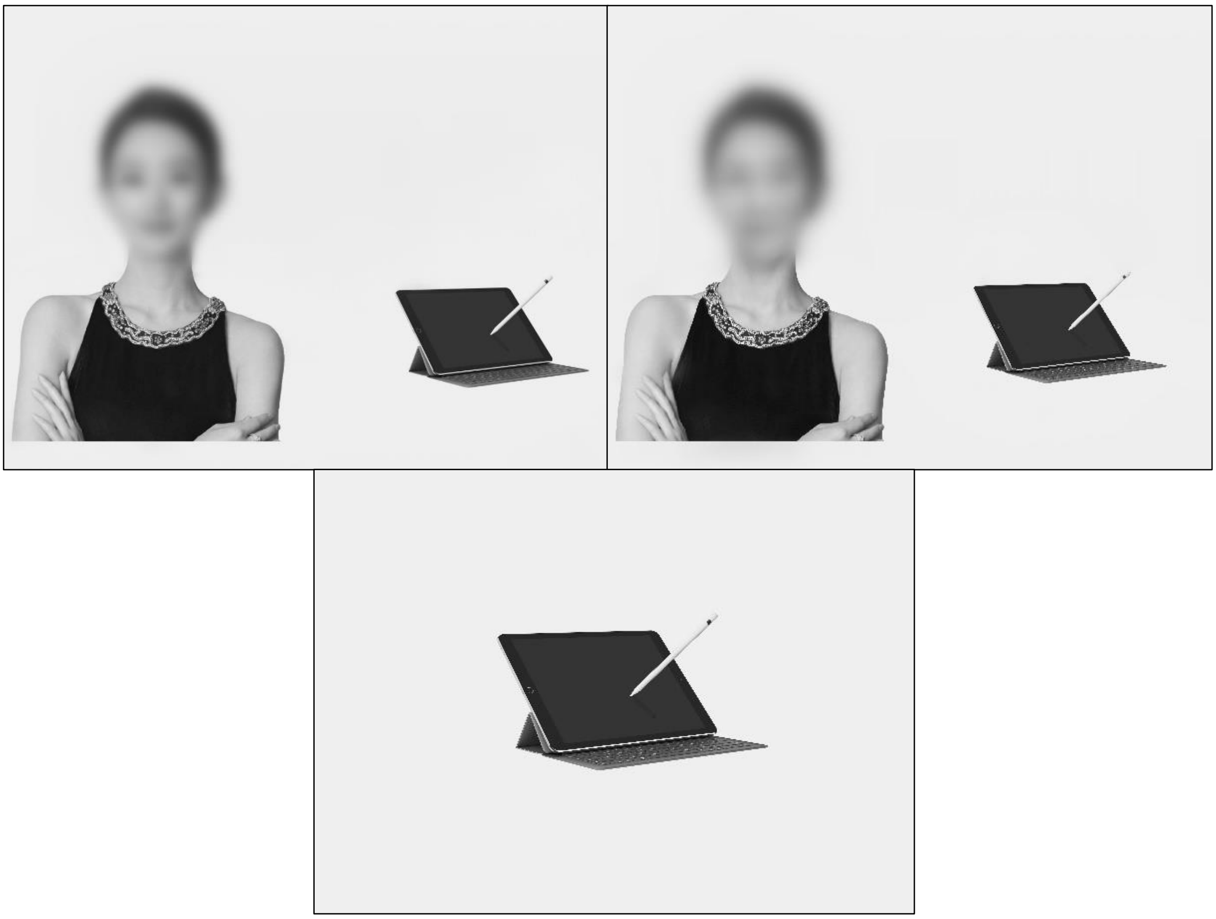

Study 3

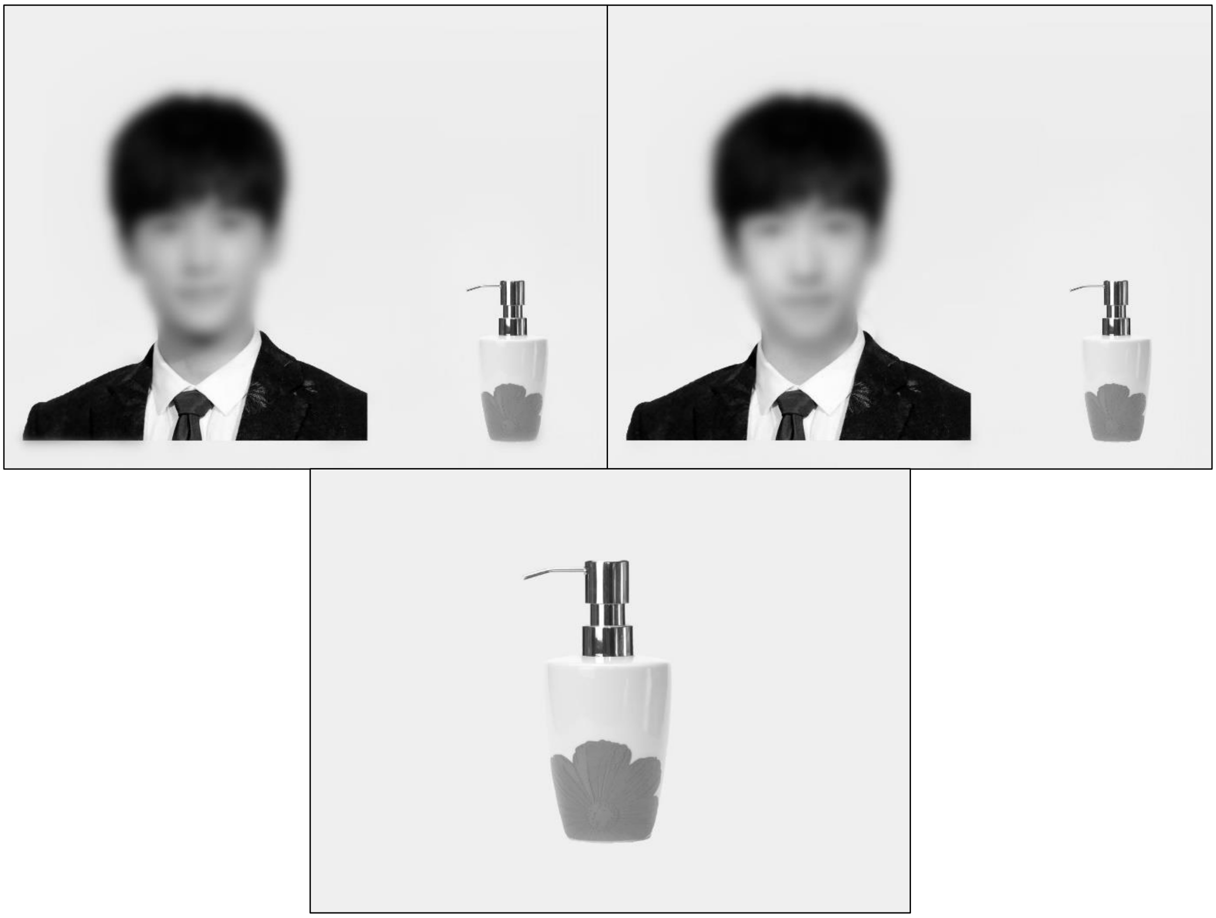

Study 4

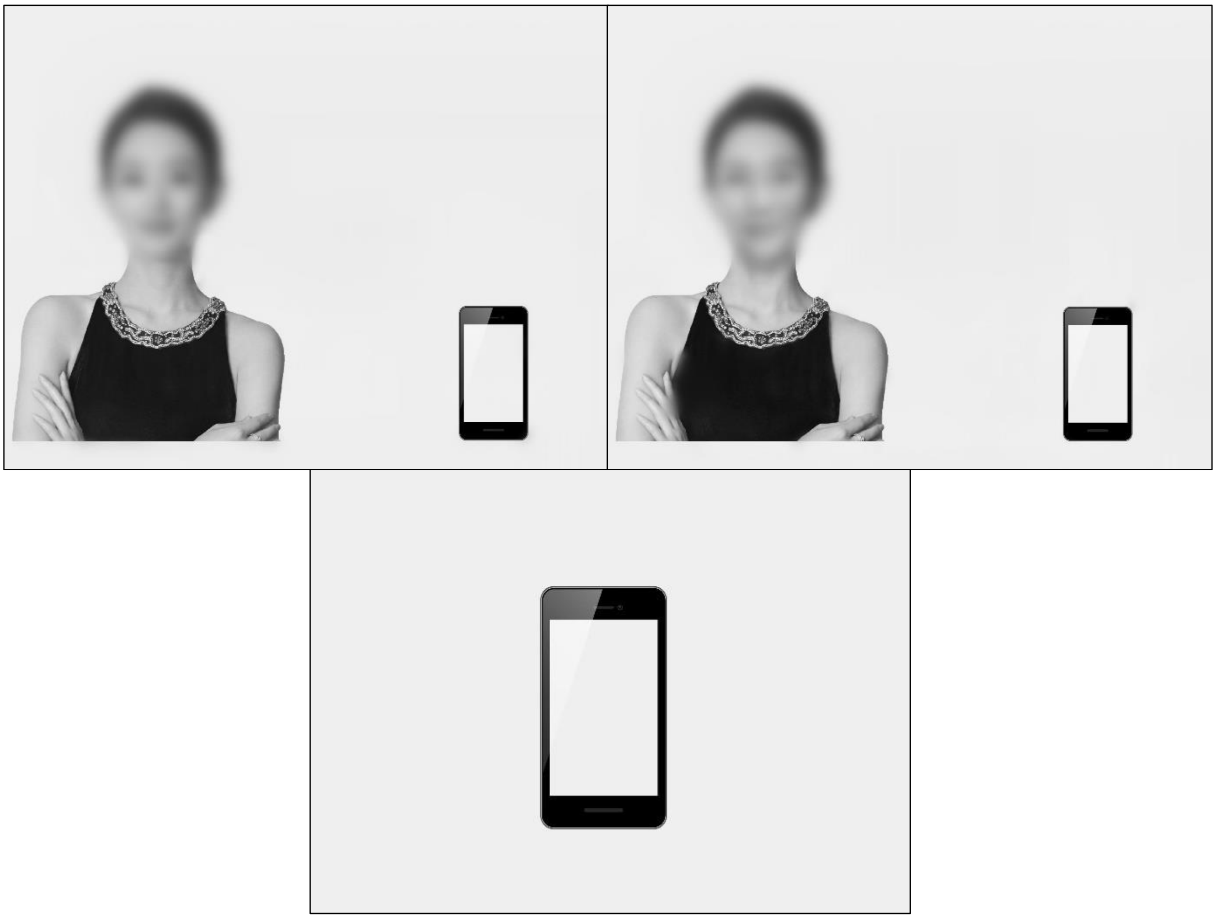

Supplement: Supplementary file 1 [file Data_Sheet_1.pdf]
